# Heterostructure Engineering of a Reverse Water Gas Shift Photocatalyst

**DOI:** 10.1002/advs.201902170

**Published:** 2019-10-04

**Authors:** Hong Wang, Jia Jia, Lu Wang, Keith Butler, Rui Song, Gilberto Casillas, Le He, Nazir P. Kherani, Doug D. Perovic, Liqiang Jing, Aron Walsh, Roland Dittmeyer, Geoffrey A. Ozin

**Affiliations:** ^1^ Key Laboratory of Functional Polymer Materials of the Ministry of Education Institute of Polymer Chemistry College of Chemistry Nankai University Tianjin 300071 P. R. China; ^2^ Materials Chemistry and Nanochemistry Research Group Solar Fuels Cluster Departments of Chemistry University of Toronto 80 St. George Street Toronto ON M5S3H6 Canada; ^3^ Department of Materials Science and Engineering University of Toronto 184 College Street Toronto ON M5S3E4 Canada; ^4^ Institute of Functional Nano & Soft Materials (FUNSOM) Jiangsu Key Laboratory for Carbon‐Based Functional Materials & Devices Soochow University 199 Ren'ai Road Suzhou Jiangsu P. R. China; ^5^ SciML, Scientific Computing Department, Rutherford Appleton Laboratory Didcot OX110QX UK; ^6^ UOW Electron Microscopy Centre, University of Wollongong Wollongong New South Wales 2500 Australia; ^7^ Key Laboratory of Functional Inorganic Material Chemistry Ministry of Education School of Chemistry and Materials Science International Joint Research Center for Catalytic Technology Heilongjiang University Harbin 150080 P. R. China; ^8^ Department of Materials, Imperial College London Exhibition Road London SW7 2AZ UK; ^9^ Department of Materials Science and Engineering, Yonsei University Seoul 03722 Korea; ^10^ Institute for Micro Process Engineering, Karlsruhe Institute of Technology Hermann‐von‐Helmholtz‐Platz 1 76344 Eggenstein‐Leopoldshafen Germany

**Keywords:** charge transfer, CO_2_ conversion, heterostructures, photocatalysts, semiconductors

## Abstract

To achieve substantial reductions in CO_2_ emissions, catalysts for the photoreduction of CO_2_ into value‐added chemicals and fuels will most likely be at the heart of key renewable‐energy technologies. Despite tremendous efforts, developing highly active and selective CO_2_ reduction photocatalysts remains a great challenge. Herein, a metal oxide heterostructure engineering strategy that enables the gas‐phase, photocatalytic, heterogeneous hydrogenation of CO_2_ to CO with high performance metrics (i.e., the conversion rate of CO_2_ to CO reached as high as 1400 µmol g cat^−1^ h^−1^) is reported. The catalyst is comprised of indium oxide nanocrystals, In_2_O_3−_ *_x_*(OH)*_y_*, nucleated and grown on the surface of niobium pentoxide (Nb_2_O_5_) nanorods. The heterostructure between In_2_O_3−_ *_x_*(OH)*_y_* nanocrystals and the Nb_2_O_5_ nanorod support increases the concentration of oxygen vacancies and prolongs excited state (electron and hole) lifetimes. Together, these effects result in a dramatically improved photocatalytic performance compared to the isolated In_2_O_3−_ *_x_*(OH)*_y_* material. The defect optimized heterostructure exhibits a 44‐fold higher conversion rate than pristine In_2_O_3−_ *_x_*(OH)*_y_*. It also exhibits selective conversion of CO_2_ to CO as well as long‐term operational stability.

## Graphical abstract

A heterostructure strategy involving two nanostructured metal oxide semiconductors is introduced to provide a new kind of highly efficient and stable photocatalyst for converting gaseous CO_2_ to CO.

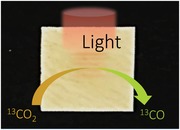

Global energy demands and climate change have stimulated intense research on the sustainable transformation of greenhouse gas CO_2_ to chemicals and fuels.^1^, ^2^, ^3^, ^4^, ^5^, ^6^ Among various approaches that enable these CO_2_ conversions, of particular interest is the gas‐phase solar powered heterogeneous catalytic reduction of CO_2_ to CO, dubbed the photocatalytic reverse water gas shift reaction. This process can enable very high CO_2_ conversion rates, and is compatible with existing chemical and petrochemical industry infrastructure, implying that it can be easily scaled, integrated, and commercialized.^7^ Key to achieving the practical implementation of this promising renewable‐energy technology is to develop efficient, robust, and scalable photocatalysts for the hydrogenation of CO_2_. Despite tremendous efforts, it remains a great challenge since the CO_2_ molecule is fully oxidized and extremely stable.

Recently, semiconducting metal oxides have been widely investigated as photocatalysts for CO2 hydrogenation mainly because of their photochemical stability. However, limited by their performance, much effort has been devoted to enhancing their photocatalytic activity.^8^, ^9^, ^10^, ^11^, ^12^ In this context, Indium oxide is one of the most widely used, n‐type transparent conducting metal oxides in electronic, optoelectronic, and optical devices due to its large optical bandgap, low electrical resistivity, and excellent photostability.^13^ Our group has recently reported that indium oxide, In_2_O_3−_ *_x_*(OH)*_y_*, with engineered oxygen vacancy (electron trapping) and hydroxide (hole trapping) defects, is active for the photocatalytic hydrogenation of CO_2_ to CO.^14^, ^15^ The CO_2_ conversion rate to CO however is at best 33.3 µmol g cat^−1^ h^−1^, which is too low for its practical implementation in a CO_2_ refinery.

Herein, we show that heterostructure engineering of In_2_O_3−_ *_x_*(OH)*_y_* with other metal oxide semiconductor can dramatically improve its photocatalytic activity. By this means, the CO_2_ to CO conversion rate was boosted from 33.3 µmol g cat^−1^ h^−1^ for pristine In_2_O_3−_ *_x_*(OH)*_y_* to as high as 1400 µmol g cat^−1^ h^−1^ for heterostructure engineered In_2_O_3−_ *_x_*(OH)*_y_*, a remarkable enhancement factor of 44‐fold. Such a dramatic enhancement in conversion rate is a rare event.^16^, ^17^ Furthermore, the heterostructure engineered In_2_O_3−_ *_x_*(OH)*_y_* exhibited excellent long‐term, operational photochemical durability.

The heterostructure between the two semiconductors In_2_O_3−_ *_x_*(OH)*_y_* and Nb_2_O_5_ nanorods (Figure S1, Supporting Information) was constructed by a facile, two‐step process (**Figure** **1**a). First, the In(OH)_3_@Nb_2_O_5_ precursor was prepared by growth of In(OH)_3_ on Nb_2_O_5_ in an ethanol/water mixture. Second, the desired In_2_O_3−_ *_x_*(OH)*_y_*@Nb_2_O_5_ heterostructure was formed by thermally induced dehydroxylation of In(OH)_3_@Nb_2_O_5_ at 250 °C in air for a predetermined time. To understand the physical and chemical properties of heterostructure engineered In_2_O_3−_ *_x_*(OH)*_y_*, we prepared pristine In_2_O_3−_ *_x_*(OH)*_y_* as a control reference sample. The synthetic procedure for making pristine In_2_O_3−_ *_x_*(OH)*_y_* is similar to In_2_O_3−_ *_x_*(OH)*_y_*@Nb_2_O_5_, except there is no Nb_2_O_5_ involved in the preparation process.

**Figure 1:**
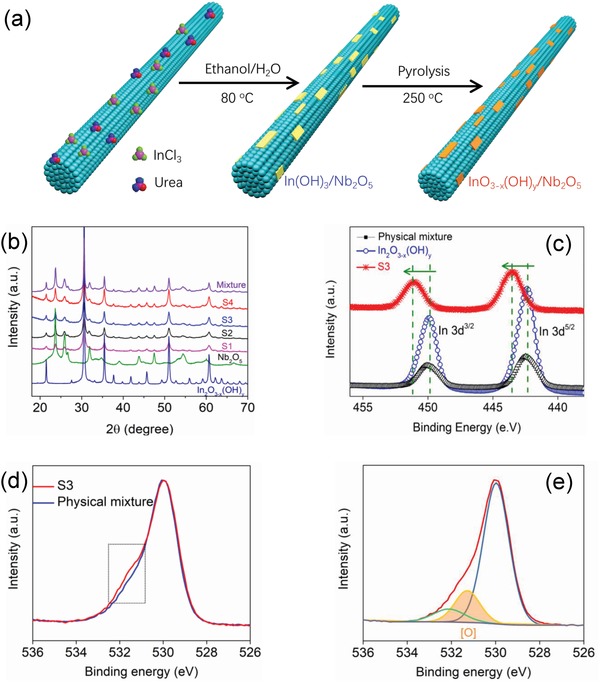
a) Illustration of the synthesis of In_2_O_3−_ *_x_*(OH)*_y_* nanocrystals grown on the surface of Nb_2_O_5_ nanorods. b) PXRD patterns of pristine In_2_O_3−_ *_x_*(OH)*_y_* and Nb_2_O_5_, a chemical heterostructure In_2_O_3−_ *_x_*(OH)*_y_*@Nb_2_O_5_. c) XPS and curve fitting for the In 3d_5/2_ and In 3d_3/2_ core level, spin–orbit split binding energies of pristine In_2_O_3−_ *_x_*(OH)*_y_*, a physical mixture In_2_O_3−_ *_x_*(OH)*_y_*/Nb_2_O_5_ and a representative chemical heterostructure In_2_O_3−_ *_x_*(OH)*_y_*@Nb_2_O_5_, S3 sample. d) O 1s XPS spectrum of S3 and physical mixture sample. e) Deconvolution of the high‐resolution O1s XPS spectrum of the S3 sample. The main peak at 530.3 eV is attributed to indium oxide. The two additional shoulder peaks at 531.7 and 532.5 eV are attributed to oxygen vacancies in the structure and surface OH groups, respectively.

Thermogravimetric analysis showed that the behavior of supported In(OH)_3_ in In(OH)_3_@Nb_2_O_5_ is similar to In(OH)_3_ alone (Figures S2 and S3, Supporting Information). One observes a sharp weight loss after the temperature reached ≈200 °C, accompanied by an endothermic peak at ≈240 °C, which corresponds to the conversion of cubic In(OH)_3_ into cubic, defect‐laden bixbyite In_2_O_3−_ *_x_*(OH)*_y_* through controlled dehydroxylation of the number of hydroxyl groups with concomitant loss of water. A set of three different In_2_O_3−_ *_x_*(OH)*_y_* @Nb_2_O_5_ heterostructure samples were fabricated from the corresponding precursor In(OH)_3_@Nb_2_O_5_ (Figure S4, Supporting Information). These samples are denoted S1–S4, where the label 1–4 refers to samples with gradually increasing In_2_O_3−_ *_x_*(OH)*_y_* content. The amount of In_2_O_3−_ *_x_*(OH)*_y_* in S1–S4 were 9.79%, 15.49%, 23.21%, and 30.7%, respectively, as quantified by inductively coupled plasma‐atomic emission spectroscopy, For comparison, a physically blended mixture of nanocrystalline In_2_O_3−_ *_x_*(OH)*_y_* and Nb_2_O_5_ nanorods was also prepared with the content of In_2_O_3−_ *_x_*(OH)*_y_* in the mixture adjusted to be identical with that in S3.

Powder X‐ray diffraction (PXRD) peaks of In_2_O_3−_ *_x_*(OH)*_y_*@Nb_2_O_5_, pristine In_2_O_3−_ *_x_*(OH)*_y_*, and Nb_2_O_5_ are shown in Figure 1b, all of the peaks of In_2_O_3−_ *_x_*(OH)*_y_* in S1–S4 were matched well with pristine In_2_O_3−_ *_x_*(OH)*_y_*, indicating that the In_2_O_3−_ *_x_*(OH)*_y_*@Nb_2_O_5_ are successfully prepared after calcining the precursor of In(OH)_3_@Nb_2_O_5_, at the right temperature for the appropriate time. To explore the effect of the heterostructure on the electronic structure of In_2_O_3−_ *_x_*(OH)*_y_*, X‐ray photoelectron spectroscopy (XPS) of the S3, pristine In_2_O_3−_ *_x_*(OH)*_y_*, and the physical mixture were conducted (Figure 1c, and Figure S5, Supporting Information). The In 3d core level spectra of pristine In_2_O_3−_ *_x_*(OH)*_y_* and the physical mixture show two peaks located at 442.8 and 450.3 eV, which are attributed to the characteristic spin–orbit split 3d_5/2_ and 3d_3/2_, respectively. Interestingly, the 3d_5/2_ and 3d_3/2_ peaks of In_2_O_3−_ *_x_*(OH)*_y_* in the S3 sample are shifted toward higher binding energy, located at 443.3 and 450.9 eV, respectively (Figure 1c). This indicates an increase in effective positive charge of the In sites as a result of the heterostructure engineering. The increased population of coordinately unsaturated In sites arising from the larger population of oxygen vacancies seen in the O1s photoemission spectra in In_2_O_3−_ *_x_*(OH)*_y_* can be related to the enhanced binding energy. This point was further supported by XPS analysis of O species in these samples. Considering the content of In_2_O_3−_ *_x_*(OH)*_y_* in S3 and physical mixture samples is identical, we compared the oxygen vacancy [O] concentrations in the two samples. As shown in Figure 1d,e, the population of [O] in the S3 was 20.9%, which is higher than that in physical mixture sample (16.6%) (Figure S6, Supporting Information). Later in this article, we demonstrate that such an increase of oxygen vacancies in heterostructure engineered In_2_O_3−_ *_x_*(OH)*_y_* enhances the population and lifetime of photoexcited electron–hole pairs and as a result provides a boost to the photocatalytic activity.^18^, ^19^

We took S3 sample as representative to examine its morphology and chemical heterostructure using high resolution transmission electron microscopy (HRTEM). From **Figure** **2**a, In_2_O_3−_ *_x_*(OH)*_y_* nanocrystals with a diameter of ≈4 nm, uniformly distributed on the surface of Nb_2_O_5_, are observable. It is noted that the use of ammonia rather than urea as the base hydrolysis source in the synthesis, proved unable to produce heterostructures of In_2_O_3−_ *_x_*(OH)*_y_* and Nb_2_O_5_. Instead, only two separate normal phases of In_2_O_3−_ *_x_*(OH)*_y_* and Nb_2_O_5_ resulted (Figure S7, Supporting Information). This observation demonstrates the importance of the controlled nucleation and growth process for making heterostructured In_2_O_3−_ *_x_*(OH)*_y_*@Nb_2_O_5_ by using the strategy of slow release of base by the decomposition of urea at 80 °C. The HRTEM data show well‐defined heterojunctions between nanocrystalline In_2_O_3−_ *_x_*(OH)*_y_* and Nb_2_O_5_ nanorods (Figure 2b). Analysis of the In_2_O_3−_ *_x_*(OH)*_y_* nanocrystals on the Nb_2_O_5_ nanorods in the HRTEM image reveals a lattice spacing of 4.03 Å corresponding to the (211) plane. Figure 2c–f shows the scanning transmission electron microscopy (STEM) image and corresponding elemental mappings of Nb, In, and O, respectively. It can be clearly seen that In_2_O_3−_ *_x_*(OH)*_y_* was uniformly dispersed on the surface of Nb_2_O_5_ nanorod, a favorable requirement for optimized catalytic activity.

**Figure 2:**
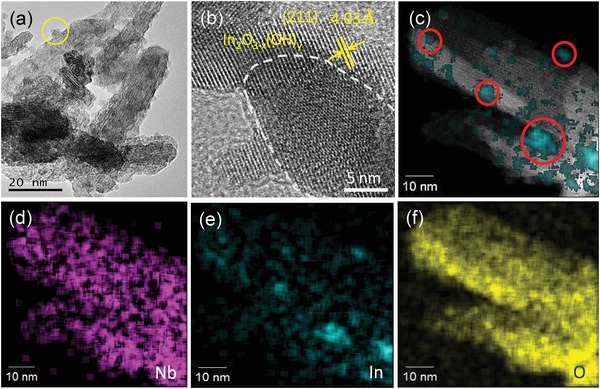
a) Low‐resolution TEM image of S3. Yellow circle indicates that In_2_O_3−_ *_x_*(OH)*_y_* nanocrystal with a diameter of ≈4 nm was grown on the surface of Nb_2_O_5_ nanorod. b) HRTEM images of S3. c–f) STEM image and corresponding EFTEM elemental (Nb, In, O) mapping.

All samples exhibited a mesoporous structure (Figure S8, Supporting Information), and the Brunauer–Emmett–Teller (BET) specific surface areas of Nb_2_O_5_, S1, S2, S3, and S4 are 84.6, 63.7, 60, 88.8, and 96.6 m^2^ g^−1^, respectively, were lower than that of pristine In_2_O_3−_ *_x_*(OH)*_y_* (117 m^3^ g^−1^) (Figure S9 and Table S1, Supporting Information). Noted also, is that the BET surface areas of S1 and S2 are lower than that of Nb_2_O_5_. This can best be explained by the fact that nanocrystalline In_2_O_3−_ *_x_*(OH)*_y_* grown within the mesopores of Nb_2_O_5_ nanorods, decreases the BET surface areas of S1 and S2 compared to Nb_2_O_5_. With further increase of the content of In_2_O_3−_ *_x_*(OH)*_y_* in S3, the mesoporous structure of In_2_O_3−_ *_x_*(OH)*_y_* improves their surface areas. This result, together with those from the TEM observations can explain the growth process of In_2_O_3−_ *_x_*(OH)*_y_* nanocrystal on the surface of Nb_2_O_5_ to form heterostructures. First, the indium chloride and urea were adsorbed in the mesopores of Nb_2_O_5_. Second, In(OH)_3_ precursor forms and anchors to the surface of Nb_2_O_5_ concomitant with the decomposition of urea to NH_3_ at 80 °C. Finally, In_2_O_3−_ *_x_*(OH)*_y_* nanocrystals form on the surface of Nb_2_O_5_ nanorods during the dehydroxylation process at 250 °C.

The mesoporous structure of the as‐synthesized photocatalysts (**Figure** **3**a) is advantageous for fast diffusion of CO_2_ to catalytic centers thereby improving their performance. Photocatalytic activity was measured using the as‐synthesized samples deposited on borosilicate filter films (Figure 3b). To ensure the products did not originate from adventitious carbon residues in our samples, isotope labeled ^13^CO_2_ authenticated the origin of the products of the reduction reaction.

**Figure 3:**
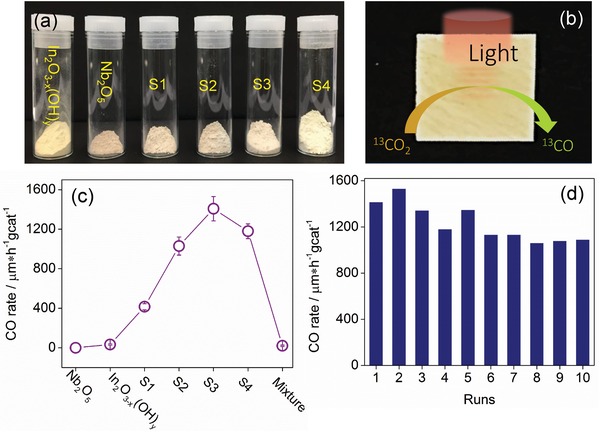
a) Digital photographs of as‐prepared pristine In_2_O_3−_ *_x_*(OH)*_y_*, S1‐S4, and Nb_2_O_5_. b) Digital photograph of S3 sample deposited on borosilicate filter film for photocatalytic measurement. c) Photocatalytic performance of the listed materials. d) Long‐term photocatalyst stability measurements of the S3 sample.

We observed a remarkable enhancement of the hydrogenation rate of CO_2_ to CO on passing from S1 to S3 (Figure 3c), reaching as high as 1400 µmol g cat^−1^ h^−1^ for S3, which then decreases for S4. This can be explained by the fact that there is more isolated In_2_O_3−_ *_x_*(OH)*_y_* in S4 rather than heterostructures of In_2_O_3−_ *_x_*(OH)*_y_*/Nb_2_O_5_. The product is completely ^13^C labeled ^13^CO without impurities (Figure S10, Supporting Information), demonstrating high selectivity of our photocatalysts for solar powered hydrogenation of CO_2_ to CO.

Note that Nb_2_O_5_ is highly inactive under these photocatalytic‐operating conditions, showing no activity toward CO_2_ to CO reduction. We also showed there is no CO produced in the dark, confirming the photocatalytic nature of the samples. It is observed that the hydrogenation rate of CO_2_ to CO over pristine In_2_O_3−_ *_x_*(OH)*_y_* and their physical mixture are only 33 and 21 µmol g_cat_^−1^ h^−1^ respectively under the same conditions, much lower than that of the heterostructured samples. These observations strongly support the proposition that heterostructure engineering of photocatalysts described herein provides a new and effective strategy for enhancing the performance of gas‐phase heterogeneous CO_2_ hydrogenation.

The key for practical applications is the long‐term stability of a photocatalyst. As shown in Figure 3b, stability testing of S3 for photocatalytic hydrogenation of CO_2_ to CO showed no decay even after 10 cycles, which involved continuous operations for 40 h. This result confirms that heterostructured In_2_O_3−_ *_x_*(OH)*_y_* is stable under operating conditions employed in this study.

In metal oxides, both bulk and surface oxygen vacancies can act as traps for photoexcited electrons, while surface hydroxyl groups can function as traps for photoexcited holes.^20^ Efficient electron–hole pair separation is a prerequisite for high performance photocatalysis, and a reaction must have sufficient time to occur before recombination to the electronic ground state. To delve more deeply into the effect of heterostructure engineering on electron–hole separation efficiency, transient state surface photovoltage (TSPV) measurements were undertaken. Details of these TSPV experiments are included in the Supporting Information. We used two different excitation energies corresponding to above bandgap (355 nm) and near‐bandgap (532 nm) excitation to understand how photoexcited charge carriers with different energies interact with the defect states available within the samples. The TSPV spectra measured in air are in **Figure** **4**. They depict the photovoltage responses to be positive for all samples under pulsed laser excitation at 355 nm (Figure 4a) and 532 nm (Figure S11, Supporting Information).

**Figure 4:**
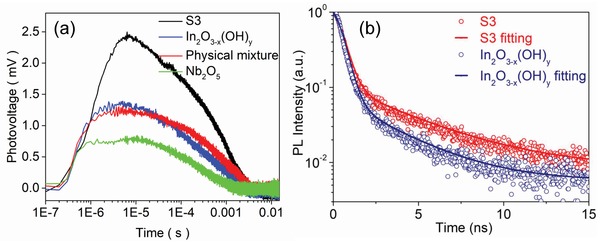
a) Transient state surface photovoltage responses of S3, pristine In_2_O_3−_ *_x_*(OH)*_y_*, and a physical mixture of In_2_O_3−_ *_x_*(OH)*_y_* and Nb_2_O_5_ nanorods, using 355 nm laser excitation. b) Fluorescence decay temporal profiles measured and fit for S3 and pristine In_2_O_3−_ *_x_*(OH)*_y_*.

The results obtained under ambient conditions indicate that adsorbed O_2_ on the surface of the samples capture photogenerated electrons, while the photogenerated holes preferentially diffuse to the collector electrode surface.^21^ Noticeably, S3 exhibits a much higher photovoltage compared to the other samples, indicating that more holes accumulate on the surface of In_2_O_3−_ *_x_*(OH)*_y_*@Nb_2_O_5_ with a noticeably longer lifetime of ≈3 ms. As mentioned earlier, XPS results for the core level oxygen demonstrate that heterostructured In_2_O_3−_ *_x_*(OH)*_y_*@Nb_2_O_5_ creates more oxygen vacancies than pristine In_2_O_3−_ *_x_*(OH)*_y_*. Thus, the larger population of oxygen vacancies in In_2_O_3−_ *_x_*(OH)*_y_* for S3 capture the photogenerated electrons, while the holes are captured by the hydroxide, thus enhancing electron–hole separation efficiency and photocatalytic activity.

Additional evidence for the enhanced electron–hole separation efficiency of S3 seen by transient state surface photovoltage measurements derives from time‐resolved fluorescence spectroscopy measurements of S3, also shown in Figure 4b. The peak of the steady‐state fluorescence of S3 is located at 410 nm (Figure S12, Supporting Information). This emission is associated with the recombination of the photogenerated hole trapped at mid‐gap hydroxide defects with the photogenerated electron trapped at sub‐bandgap O vacancy defects in In_2_O_3−_ *_x_*(OH)*_y_*.^22^ The radiative lifetime was extracted by exponential fitting using the Levenberg–Marquardt method:^23^ *y* (*t*) = *y*_0_  + *A*_1_exp (*t*/τ_1_) + *A*_2_exp (*t*/τ_2_), where τ_1_ and τ_2_ are the decay times, of the time‐resolved fluorescence decay curves for S3 (τ_1_ and τ_2_ are 0.38 and 4.05 ns, respectively), which are longer than in pristine In_2_O_3−_ *_x_*(OH)*_y_* (τ_1_ and τ_2_ are 0.35 and 3.12 ns, respectively). These results provide additional evidence for enhanced electron–hole separation in the heterostructure In_2_O_3−_ *_x_*(OH)*_y_*@Nb_2_O_5_.

At the interface between the In_2_O_3−_ *_x_*(OH)*_y_* and Nb_2_O_5_ semiconductors, one expects charge‐transfer and the formation of a space charge region to occur as the Fermi level is equilibrated across the sample. This electronic structure model is substantiated by measurement of the Fermi levels of S3, pristine In_2_O_3−_ *_x_*(OH)*_y_* and Nb_2_O_5_ nanorods at −4.03, −3.84, and −4.46 eV, respectively, by means of ultraviolet photoelectron spectroscopy, UPS (Figure S13, Supporting Information). The UPS results provide evidence for an electronic band model in which electrons flow from Nb_2_O_5_ to In_2_O_3−_ *_x_*(OH)*_y_* across the heterostructure in S3. The heterojunction alignment whereby electrons flow from Nb_2_O_5_ to In_2_O_3−_ *_x_*(OH)*_y_* is also confirmed through band alignment calculations (Figure S14, Supporting Information). Oxygen vacancies formed in the heterostructures of In_2_O_3−_ *_x_*(OH)*_y_*@Nb_2_O_5_ in S3 serve as reservoirs for photoexcited electrons, while the hydroxides act as reservoirs for photoexcited holes. The real space separation of the charge carriers stabilizes the photoexcited state, resulting in longer carrier lifetimes in In_2_O_3−_ *_x_*(OH)*_y_*@Nb_2_O_5_ that provide a significant boost in photocatalytic activity for the solar powered reverse water gas shift reaction CO_2_ + H_2_ → CO + H_2_O.

From a scientific perspective, challenges remain in the quest to understand how heterostructures between metal oxide semiconductors control photocatalytic performance. These challenges include disentangling the effects of heterostructure interfaces and interfacial charge transfer on defect populations; how these affect the lifetimes of photoexcited electron–hole pairs; the effect on the acidity and basicity of surface frustrated Lewis pairs, and their corresponding activity toward the hydrogenation of carbon dioxide. In terms of practical applications, significant improvements in conversion and energy efficiency together with a techno‐economic life cycle assessment will be required before the class of photocatalysts described in this paper is ready for engineering a pilot scale solar fuels process.

## Conflict of Interest

The authors declare no conflict of interest.

## Supporting information

Supplementary
