# Supplementary material for: Heterostructure Engineering of a Reverse Water Gas Shift Photocatalyst
Source: Adv Sci (Weinh). 2019 Oct 4;6(22):1902170. doi: 10.1002/advs.201902170 (PMC6864495; doi:10.1002/advs.201902170)
Supplement: Supplementary file 1 — Supplementary [file ADVS-6-1902170-s001.pdf]

Copyright WILEY-VCH Verlag GmbH & Co. KGaA, 69469 Weinheim, Germany, 2019.

ADVANCED SCIENCE
Open Access

Supporting Information

for *Adv. Sci.,* DOI: 10.1002/advs.201902170

Heterostructure Engineering of a Reverse Water Gas Shift Photocatalyst

Hong Wang, Jia Jia, Lu Wang, Keith Butler, Rui Song, Gilberto Casillas, Le He, Nazir P. Kherani, Doug D. Perovic, Liqiang Jing, Aron Walsh, Roland Dittmeyer, and Geoffrey A. Ozin**

Supplementary Information

Heterojunction Engineering of a Reverse Water Gas Shift Photocatalyst

Hong Wang[1], Jia Jia[2,3], Lu Wang[2,4], Keith Butler[5], Rui Song[2], Gilberto Casillas[6], Le He[4], Nazir P. Kherani[3], Doug D. Perovic[3], Liqiang Jing[7], Aron Walsh[8], Roland Dittmeyer[9], Geoffrey A Ozin[2]**

[1] Prof. H. Wang, Key Laboratory of Functional Polymer Materials of the Ministry of Education, Institute of Polymer Chemistry, College of Chemistry, Nankai University, Tianjin, 300071, P. R. China;
[2] Dr. L. Wang, Dr. J. Jia, R. Song, Prof. G. A. Ozin
Materials Chemistry and Nanochemistry Research Group, Solar Fuels Cluster, Departments of Chemistry, University of Toronto, 80 St. George Street, Toronto, Ontario M5S3H6, Canada;
[3] Dr. J. Jia, Prof. N. P. Kherani, Prof. D. D. Perovic
Department of Materials Science and Engineering, University of Toronto, 184 College Street, Toronto, Ontario M5S3E4, Canada;
[4] Dr. L. Wang, Prof. L. He
Institute of Functional Nano & Soft Materials (FUNSOM), Jiangsu Key Laboratory for Carbon-Based Functional Materials & Devices, Soochow University, 199 Ren'ai Road, Suzhou, Jiangsu, PR China;
[5] Dr. K. Butler
SciML, Scientific Computing Department, Rutherford Appleton Laboratory, Didcot, OX110QX, UK;
[6] Dr. G. Casillias
UOW Electron Microscopy Centre, University of Wollongong, Wollongong, New South Wales 2500, Australia;
[7] Prof. L. Jing
Key Laboratory of Functional Inorganic Material Chemistry, Ministry of Education School of Chemistry and Materials Science, International Joint Research Center for Catalytic Technology, Heilongjiang University, Harbin,150080, P. R. China;
[8] Prof. Aron Walsh
Department of Materials, Imperial College London, London, United Kingdom;
[9] Prof. R. Dittmeyer
Institute for Micro Process Engineering, Karlsruhe Institute of Technology, Hermann-von-Helmholtz Platz 1, 76344 Eggenstein-Leopoldshafen, Germany;

Corresponding author:

E-mail: hongwang1104@nankai.edu.cn; gozin@chem.utoronto.ca

Materials and Methods

1. Chemicals

Niobium powder (325 Mesh, Aldrich), urea (99% Aldrich), methanol (99% Aldrich), ethanol (99% Aldrich), anhydrous $InCl_3$ (99% Alpha) were used as-received without purifications.

2. Synthesis of $In_2O_{3-x}(OH)_y@Nb_2O_5$ heterostructures

2.1 Synthesis of Nb_2O_5 nanorods

Synthesis of $Nb_3O_7(OH)$ Nanorods *(S1)*: Niobium powder (680 mg) was dissolved in a hydrochloric acid solution (9 mL HCl; 10 mL deionized water) in a pyrex beaker. The aqueous solution was ultra-sonicated for 30 min and then stirred for 15 min. The solution was subsequently placed in a Teflon-lined stainless steel autoclave with 100 mL capacity. The hydrothermal reaction was performed at T = 200 °C for 24 h. After cooling to room temperature, the white product was collected through a centrifugation process and washed three times with deionized water to remove non-reacted residues. Finally, the sample was dried in a vacuum oven at T = 70 °C for 12 h. Nb_2O_5 nanorods were obtained by calcining of as-prepared $Nb_3O_7(OH)$ Nanorods at 400 ^oC at air for 6 h.

2.2 Synthesis of $In(OH)_3@Nb_2O_5$

In a typical procedure, 0.2 g of as-prepared Nb_2O_5 nanorods were dispersed in 25 mL of ethanol with sonicating for 1 h. Subsequently, a varying amount of urea dissolved in 10 mL H_2O was added, the mixed solution was further sonicated for 30 minutes. Finally, a varying amount of anhydrous $InCl_3$ (the molar ratio of urea to $InCl_3$ is fixed as 2:1) dissolved in 15 mL ethanol was added dropwise to above mixture solution and then heated at 80 °C in an oil bath under magnetic stirring for 12h. Following cooling to room temperature, the red products were collected though centrifugation and washed with water to remove non-reacted residues. The $In(OH)_3@Nb_2O_5$ precursors were obtained and dried for 48 h at ambient temperature. The added urea amounts for S1, S2 and S3 are 0.96 g, 2.88 g and 3.8 g, respectively.

2.3 Synthesis of $In_2O_{3-x}(OH)_y@Nb_2O_5$ Heterostructures

The as-synthesized $In(OH)_3@Nb_2O_5$ precursors were placed into an oven and treated at 350 °C in air for 3h to obtain the corresponding $In_2O_{3-x}(OH)_y@Nb_2O_5$ samples.

3. Methods

3.1 Characterization

Powder X-ray diffraction was performed on a Bruker D2-Phaser X-ray diffractometer, using Cu Kα radiation at 30 kV. Nitrogen adsorption isotherms were obtained at 77 K using a Quantachrome Autosorb-1-C. The surface area of each sample was determined using BET theory, and pore size distributions were determined with NLDFT. The amount of $In_2O_{3-x}(OH)_{y.}$ in heterostructures were quantified by inductively coupled plasma-atomic emission spectroscopy (ICP−AES, Thermo Electron Corp. Adv. ER/S). Before the ICP measurements, the heterostructures were immersed in concentrated nitric acid with agitation for 12 h to dissolve the $In_2O_{3-x}(OH)_y$. X-ray photoelectron spectroscopy (XPS) was performed using a PerkinElmer Phi 5500 ESCA spectrometer in an ultrahigh vacuum chamber with a base pressure of 1×10^{-9} Torr. The spectrometer uses an Al Kα X-ray source operating at 15 kV and 27 A. The samples used in XPS analyses were prepared by drop-casting aqueous dispersions onto p-doped Si (100) wafers in the case of the $In_2O_{3-x}(OH)_y$. samples. All data analyses were carried out using the Multipak fitting program, and the binding energies were referenced to the NIST-XPS database and the Handbook of X-ray Photoelectron Spectroscopy [*S2*-*S3*] The carbon peak was calibrated as 285 eV. Thermogravimetric analysis was conducted by placing approximately 6 mg of samples on TA Instruments SDT Q600 thermogravimetric analyzer/differential scanning calorimeter in an alumina pan under 100 mL/min flow of compressed air. The temperature was steadily increased from room temperature to 700 °C at a rate of 5 °C/ min. The Fermi level position with respect to the vacuum level is determined by measuring the work function of a thin film sample. The work function is determined by the binding energy of the secondary electron cut-off (SECO)

on the high binding energy side of the measured UPS spectrum. The work function is equal to the difference between photon energy and SECO binding energy. Transmission electron microscope (TEM) and high-resolution TEM (HRTEM) images were taken on a JEOL JEM-2100F transmission electron microscopy operated at 200 kV. Time-resolved fluorescence kinetics was measured on a Steady/Transient Steady State Fluorescence Spectrometer (F900, Edinburgh Instruments, UK) using Time Correlated Single Photon Counting. The samples were excited by a Pulsed Laser Diode (LDH-P-C-375, PicoQuant, Germany) at 375 nm with average power of 1mW while emissions were taken at 410 nm. The instrumental response function was 0.1 ns. The transient state surface photovoltage (TSPV) of the samples were measured in air atmosphere at room temperature. In a typical measurement, the sample chamber was connected to an ITO glass at the top electrode and a steel substrate at bottom electrode, and a thick mica spacer was placed between the ITO glass and the sample to decrease the space charge region at the ITO-sample interface. The metal is a ground electrode that is not connected to a battery. The function of which is to ensure that the state surface photovoltage at the metal side is zero. Upon light irradiation, photoinduced carriers will diffuse to the surface of the sample. Adsorbed O_2 on the surface of the samples capture photogenerated electrons, while the photogenerated holes preferentially diffuse to the collector electrode surface, which manifested a positive TPV response. The samples were excited by a laser pulse at 355 nm or 532 nm with 10 ns width from the second harmonic of a neodymium-doped yttrium aluminum garnet (Nd:YAG) laser. The signals were amplified with a pre-amplifier, and registered with a 1 GHz digital phosphor oscilloscope.

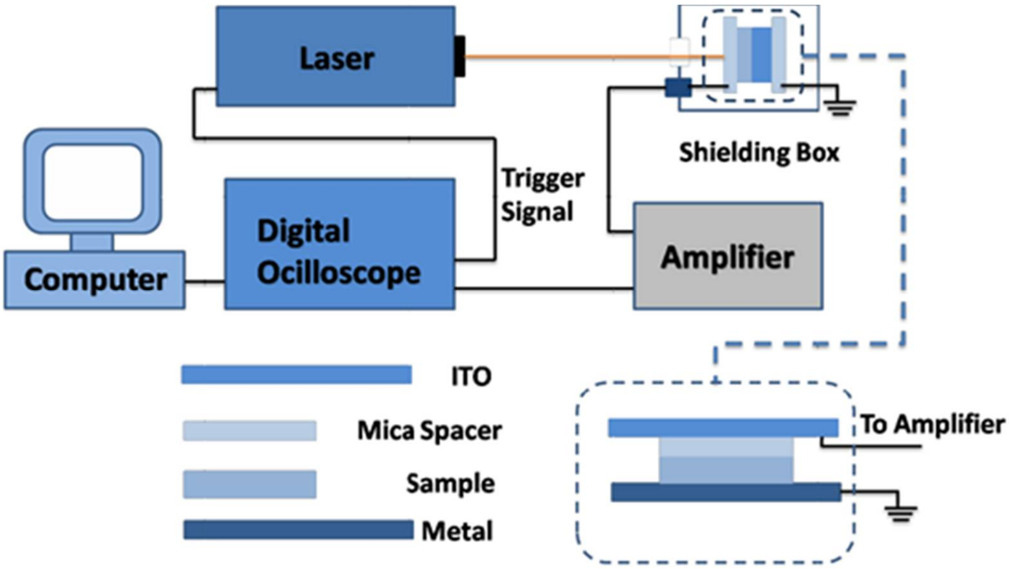

Scheme S1. Schematic of a Transient State Surface Photovoltage (TSPV) Instrument

3.2 Gas-Phase Catalytic Measurements

Gas-phase CO_2 reductive testing samples were prepared by drop casting catalysts (~3 mg) from an aqueous dispersion onto binder free borosilicate glass microfiber filters having an area of ~1.2 cm^2 (Whatman, GF/F, 0.7 μm). Two tests were then performed on catalyst samples and the duration time of each testing was 4 h: (1) in the dark at room temperature (RT), (2) under irradiation from a 300 W Xe lamp at 25 kW m^{-2}. The gas-phase catalytic measurements were conducted in a custom-built 1.8 mL stainless steel batch reactor with a fused silica view port sealed with a Viton O-ring. The reactor was evacuated using an Alcatel dry pump prior to being purged with H_2 (99.9995%) at a flow rate of 20 mL min^{-1}. After purging, the reactor was filled with H_2 and CO_2 gas at a 1:1 pressure ratio to a total pressure of 27 psi prior to being sealed. The pressure inside the reactor was monitored during the reaction using an Omega PX309 pressure transducer. Reactors were irradiated with a 300 W Xe lamp for a duration of 4 h for each testing. The spectral output from the 300 W Xe lamp was measured using a StellarNet Inc spectrophotometer and the power of the incident irradiation was measured using a Spectra-Physics Power meter (model 407A). Pressure and temperature inside the reactor after 4 h testing stabilized at 29-30 psi and 60°C, respectively. This is consistent assuming a closed system with no significant

change of the number of moles due to the reaction and validity of the ideal gas law. Note that the temperature of the catalyst is well above the gas temperature due to the photothermal effect. The sample temperature depends on the incident radiation, the design of the test cell, the operating conditions and the characteristics of the material. The sample temperature has been investigated by Raman spectroscopy for other catalyst samples prior to this work, but not measured here. Yet due to the unchanged conditions a large deviation among the temperatures of the different samples discussed here is considered unlikely. Product gases were analyzed with a flame ionization detector (FID) installed in a SRI-8610 gas chromatograph (GC) with a 6'Haysep D column. The CO formation rate shown in Fig. 3 was obtained from the measured CO content in the gas after 4 h testing by dividing with the time and the catalyst mass. Note that the so defined CO formation rate is a time-averaged value. According to kinetics and thermodynamics the CO formation rate declines with increasing conversion until it reaches zero when approaching the equilibrium. This means that the comparison of the time-averaged CO rate generally underestimates the true activity differences between the samples. The size of this effect increases with conversion. It will be particularly large if the system approaches equilibrium during testing. The experimental conversions obtained with samples S1 to S4 were much higher than calculated from the equilibrium of the reverse water gas shift reaction at the measured gas phase temperature. For example, S3 reached 3.7 % conversion, which can only be explained by a catalyst temperature of 155 °C or beyond or by an effect of the incident photons on the reaction equilibrium. Isotope tracing experiments were performed using $^{13}CO_2$ (99.9 at%; Sigma Aldrich). The reactor was evacuated prior to being injected with $^{13}CO_2$ followed by H_2. Isotopically labeled product gases were measured using an Agilent 7890A gas chromatographic mass spectrometer (GC-MS) with a 60 m GS-Carbonplot column fed to the mass spectrometer.

3.3 Electronic structure calculations

Calculations on the crystal structures were carried out under periodic boundary conditions, using the projector-augmented wave (PAW) (*S4*), pseudopotential

approach in the VASP *(S5)* code. The structures were initially relaxed using the PBEsol *(S5)* functional, with a cut-off energy of 500 eV and *k*- point mesh sampling density with a target length cut-off of 25 Å, as prescribed by Moreno and Soler *(S6)*. To obtain a quantitative electronic structure the resultant systems were then treated using the HSE06 (*S7*) functional, mixing 25% of screened exact exchange. Band alignment was performed by creating 2D slabs of each material and calculating the vacuum potential. The highest occupied and lowest unoccupied eigenvalues from bulk calculations were then placed relative to the vacuum potential, using the MacroDensity code (*S8*).

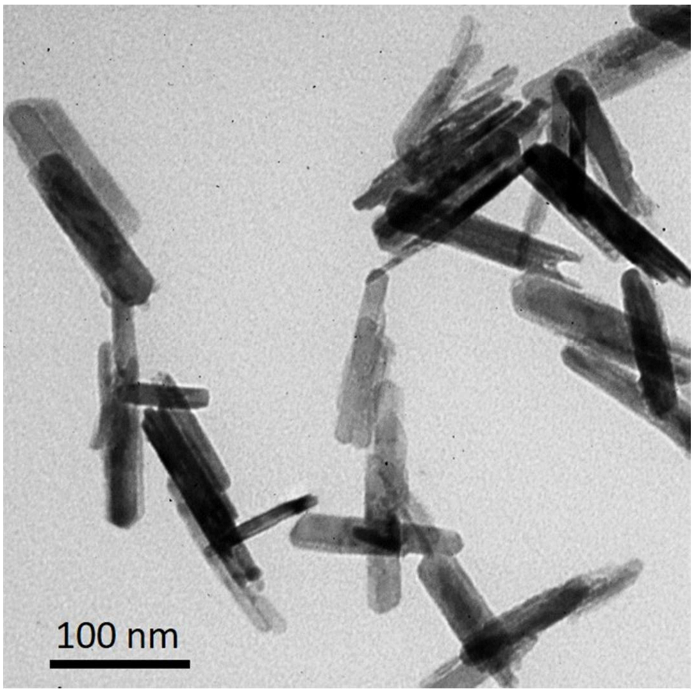

Fig. S1. TEM image of Nb_2O_5 nanorods.

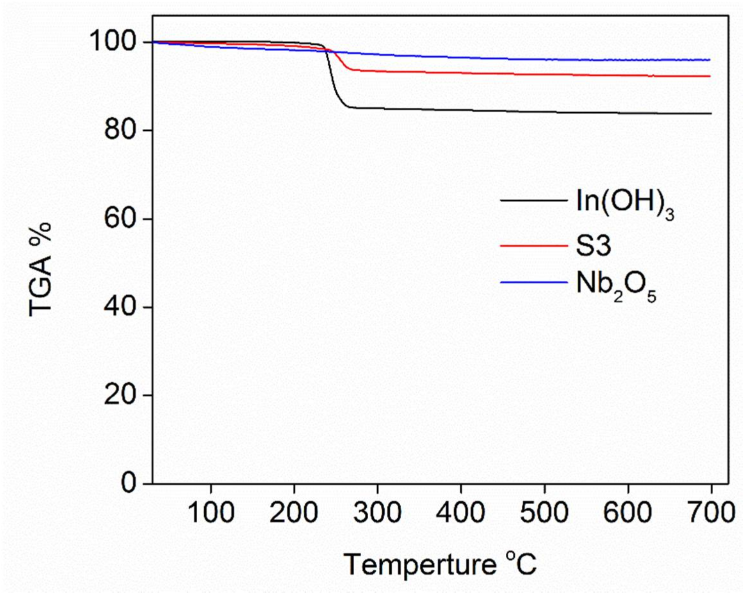

Fig. S2. Thermogravimetric analysis of $In(OH)_3$, S3 and Nb_2O_5 nanorods.

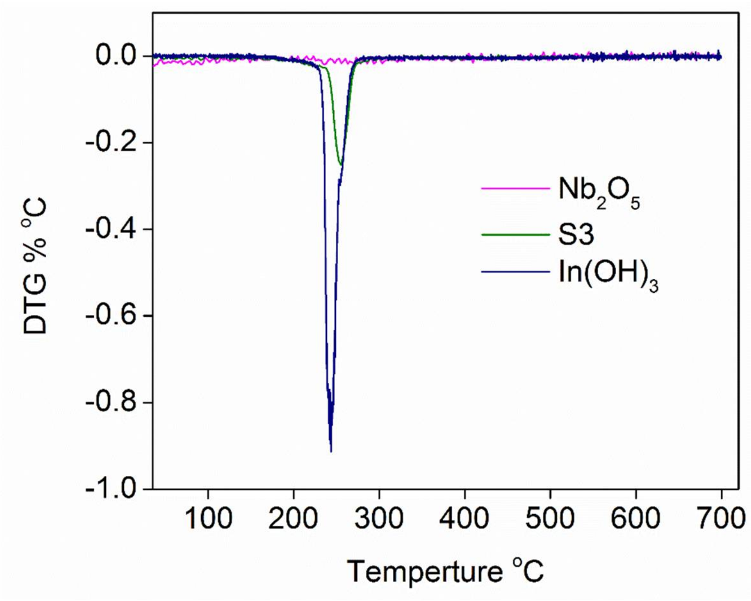

Fig. S3. Differential thermal analysis of $In(OH)_3$, S3 and Nb_2O_5 nanorods.

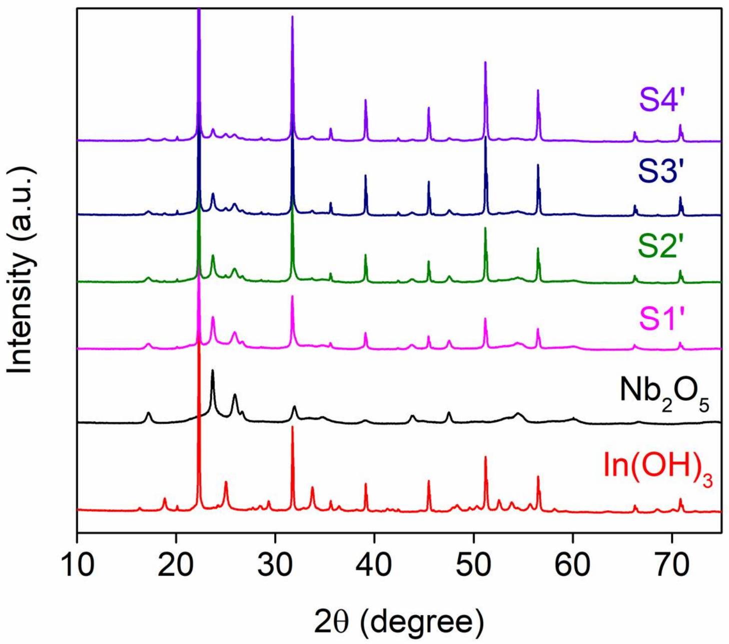

Fig. S4. PXRD patterns of pristine $In(OH)_3$, Nb_2O_5 and their heterostructures. These samples are denoted as S1'-S3', labeled with gradually increasing $In(OH)_3$ contents. It can be seen clearly that there is no PXRD peaks shifts of $In(OH)_3$ from S1' to S4' samples.

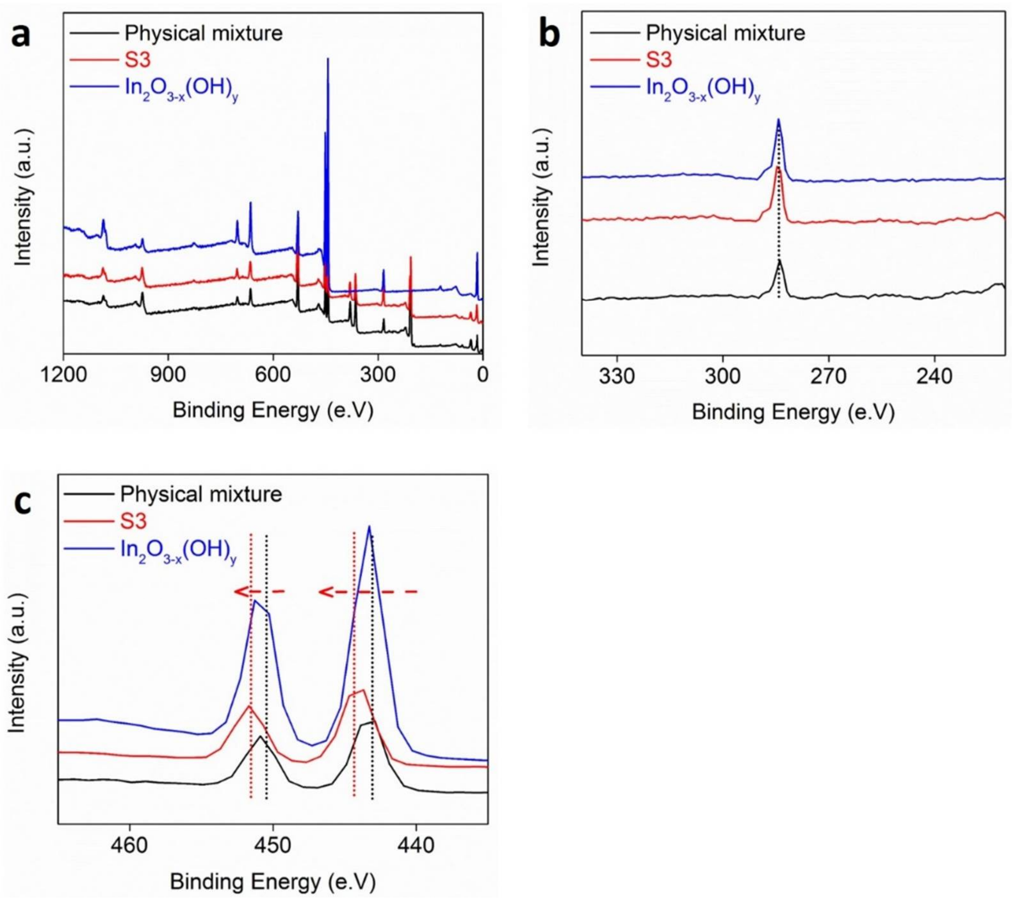

Fig. S5. XPS spectrum of pristine $In_2O_{3-x}(OH)_y$, physical mixture and S3 sample. (**a**) Full spectrum of pristine $In_2O_{3-x}(OH)_y$, S3 sample and physical mixture. (**b**) C1s (284.6 eV) spectrum which serve as reference for calibration. (**c**) Enlarged $In3d_{5/2}$ and $In3d_{3/2}$ peaks of pristine $In_2O_{3-x}(OH)_y$, physical mixture and S3 sample.

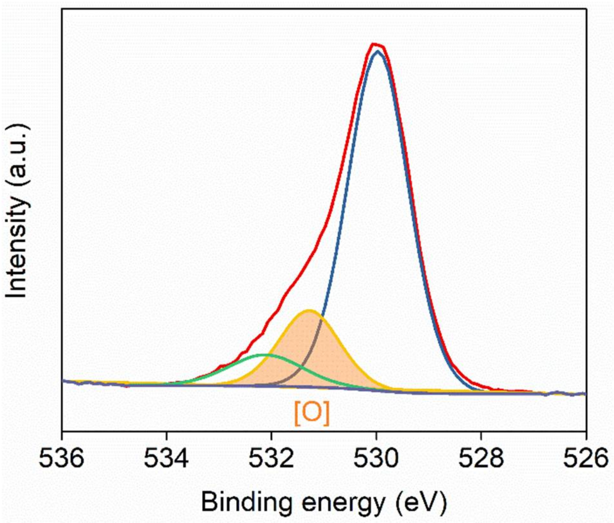

Fig. S6. High-resolution O1s XPS spectrum of physical mixture sample. The population of oxygen vacancy is 16.6%.

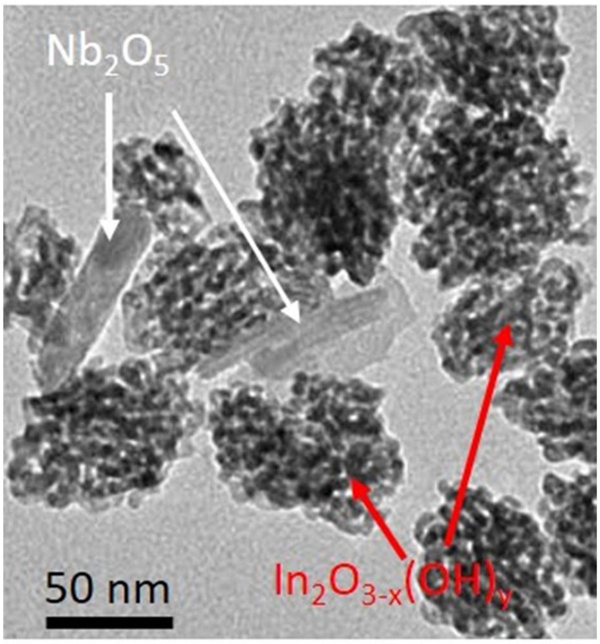

Fig. S7. TEM image of Nb_2O_5 nanorods and $In_2O_{3-x}(OH)_y$ composite prepared with ammonia method *(S9)*. It can be clearly seen that the Nb_2O_5 nanorods and $In_2O_{3-x}(OH)_y$ species are segregated in the composite.

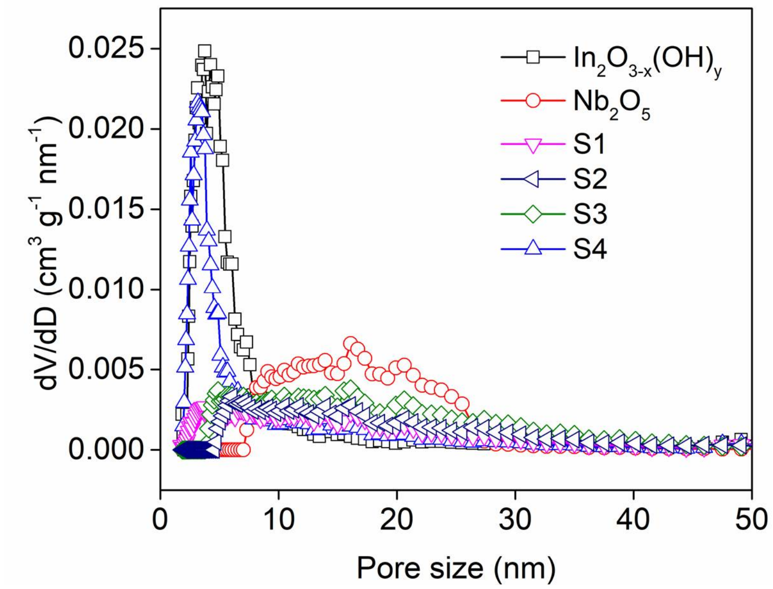

Fig. S8. Pore size distribution of $In_2O_{3-x}(OH)_y$, Nb_2O_5 and their heterostructures.

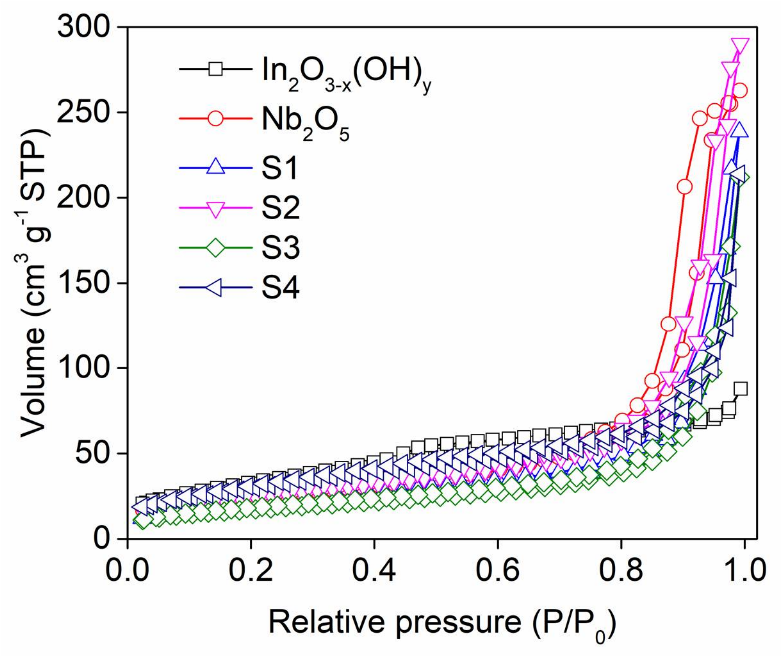

Fig. S9. N_2 absorption–desorption isotherms of $In_2O_{3-x}(OH)_y$, Nb_2O_5 and their heterostructures.

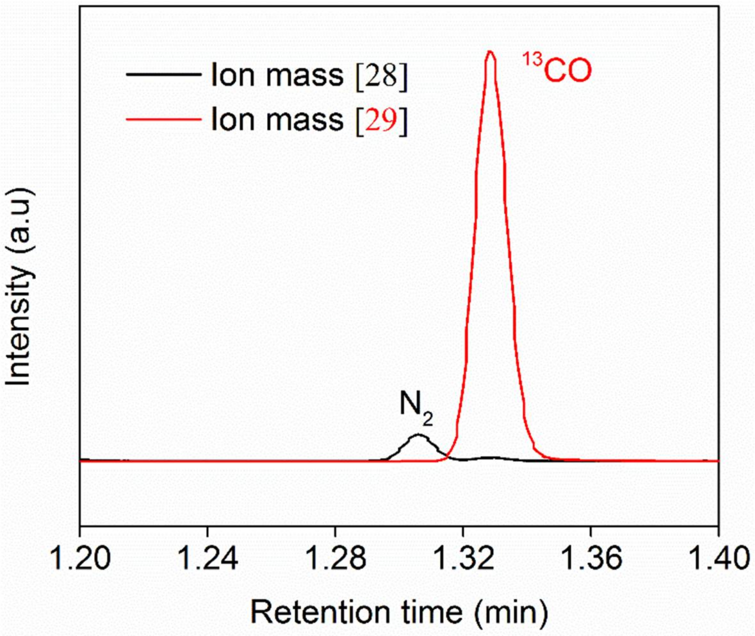

Fig. S10. Mass spectroscopy of S3 sample generated ^{13}CO from $^{13}CO_2$. The 28 AMU mass fragment peak at approximately 1.32 min corresponds to N_2 and the 29 AMU mass fragment peak at approximately 1.35 min corresponds to ^{13}CO. The fact that there is no peak near 1.35 min retention time for the 28 AMU curve shows that there is no ^{12}CO in the products generated from sources of adventitious ^{12}C.

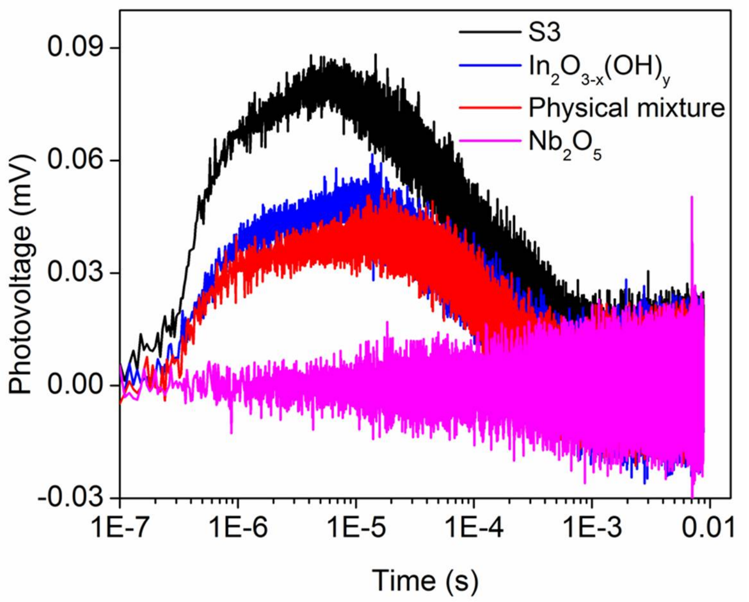

Fig. 11. Dynamics of photogenerated charge carriers. TSPV responses of S3, pristine $In_2O_{3-x}(OH)_y$, physical mixture of $In_2O_{3-x}(OH)_y$ and Nb_2O_5 nanorods under 532 nm laser excitation.

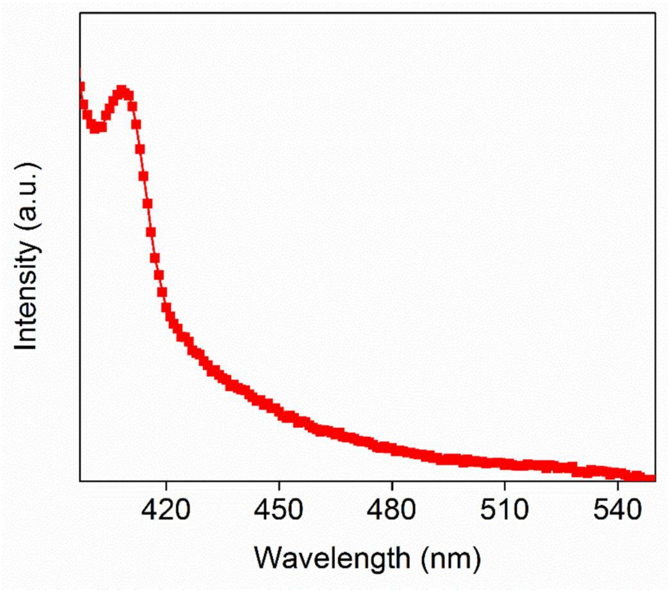

Fig. S12. Room-temperature laser induced photoluminescence spectrum of S3 excited at 325 nm. The emission at 410 nm of $In_2O_{3-x}(OH)_y$ in S3 most likely originates from recombination of photogenerated electrons trapped in oxygen vacancies and holes trapped at hydroxide groups. Note, there is no emission peak from Nb_2O_5 nanorods excited at 325 nm.

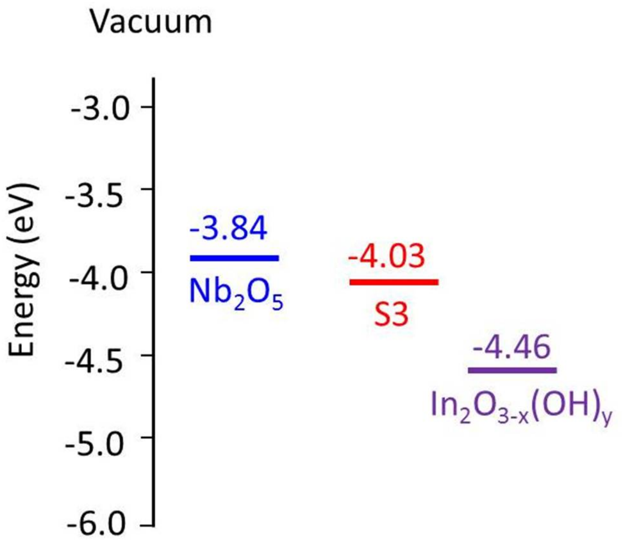

Fig. S13. Fermi energies of pristine Nb_2O_5, $In_2O_{3-x}(OH)_y$, and S3, respectively.

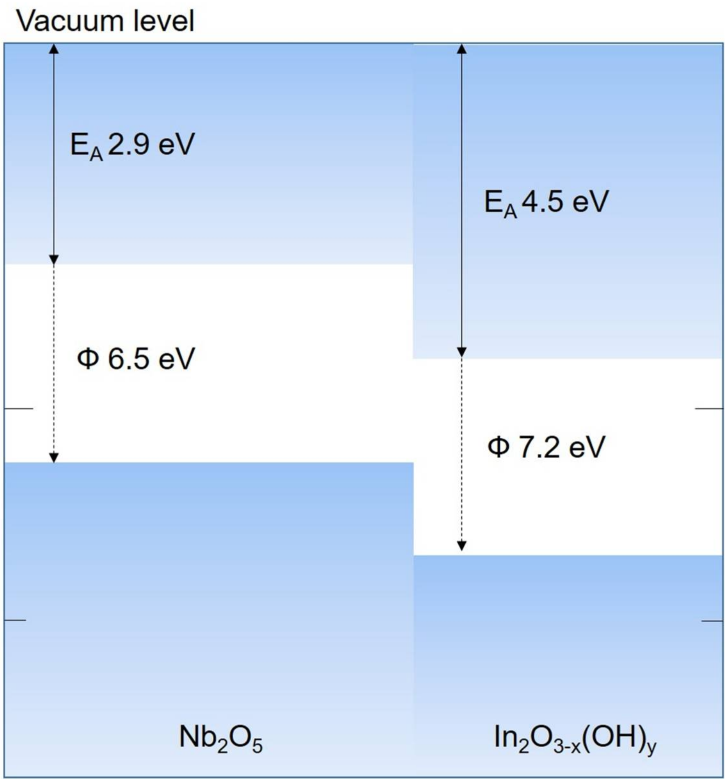

Figure S14. Calculated electronic band-alignment-diagram for $In_2O_{3-x}(OH)_y$ with Nb_2O_5.

References:

S1. Jia, J. *et al*. Visible and near-infrared photothermal catalyzed hydrogenation of gaseous CO_2 over nanostructured Pd@Nb_2O_5. *Adv. Sci.* **3**, 1600189 (2016).

S2. a) H. Fujimori, M. Kakihana, K. Ioku, S. Goto, M. Yoshimura, *Appl. Phys. Lett.* 2001, 79, 937; b) R. C. Maher, L. F. Cohen, J. C. Gallop, E. C. Le Ru, P. G. Etchegoin, *J. Phys. Chem. B* 2006, 110, 6797; c) T. A. Nachal'naya, V. D. Andreyev, E. V. Gabrusenok, Diamond Relat. Mater. 1994, 3, 1325; d) L. Han, M. Zeman, A. H. M. Smets, *Nanoscale* 2015, 7, 8389.

S3 a) L. J. Burcham, J. Datka, I. E. Wachs, *J. Phys. Chem. B* 1999, 103, 6015; b) U. Balachandran, N. G. Eror, *J. Mater. Sci. Lett.* 1982, 1, 374.

S4. Blöchl, P. E., Jepsen, O. & Andersen, O. K. Improved tetrahedron method for Brillouin-zone integrations. *Phys. Rev. B* **50**, 16223 (1994).

S5 Kresse, G. & Joubert, D. From ultrasoft pseudopotentials to the projector augmented-wave method. *Phys. Rev. B* **59**, 1758, (1999).

S6 Perdew, J. P. *et al*, Restoring the density-gradient expansion for exchange in solids and surfaces. *Phys. Rev. Lett.* **100**, 136406 (2008).

S7, Heyd, J. & Scuseria, G. E. Hybrid functionals based on a screened coulomb potential. *J. Chem. Phys.* **124**, 219906 (2006).

S8 K. T. Butler, J. Buckeridge, C. R.A. Catlow, A. Walsh, Crystal electron binding energy and surface work function control of tin dioxide. *Phys. Rev. B* **89**, 115320 (2014).

S9. Hoch, L. B. *et al*. The rational design of a single-component photocatalyst for gas-phase CO_2 reduction using both UV and visible Light. *Adv. Sci.* **1**, 1 (2014).
